# The Effect of Bee Venom Peptides Melittin, Tertiapin, and Apamin on the Human Erythrocytes Ghosts: A Preliminary Study

**DOI:** 10.3390/metabo10050191

**Published:** 2020-05-13

**Authors:** Agata Światły-Błaszkiewicz, Lucyna Mrówczyńska, Eliza Matuszewska, Jan Lubawy, Arkadiusz Urbański, Zenon J. Kokot, Grzegorz Rosiński, Jan Matysiak

**Affiliations:** 1 Department of Inorganic and Analytical Chemistry, Poznan University of Medical Sciences, 60-780 Poznan, Poland; agataswiatly@gmail.com (A.Ś.-B.); eliza.matuszewska@ump.edu.pl (E.M.); zjk@ump.edu.pl (Z.J.K.); 2 Department of Cell Biology, Faculty of Biology, Adam Mickiewicz University in Poznan, 61-614 Poznan, Poland; lumro@amu.edu.pl; 3 Department of Animal Physiology and Development, Faculty of Biology, Adam Mickiewicz University in Poznan, 61-614 Poznan, Poland; j.lubawy@amu.edu.pl (J.L.); arur@amu.edu.pl (A.U.); rosin@amu.edu.pl (G.R.)

**Keywords:** bee venom peptides, melittin, tertiapin, apamin, hemolytic activity, human erythrocytes, erythrocytes ghosts proteome

## Abstract

Red blood cells (RBCs) are the most abundant cells in the human blood that have been extensively studied under morphology, ultrastructure, biochemical and molecular functions. Therefore, RBCs are excellent cell models in the study of biologically active compounds like drugs and toxins on the structure and function of the cell membrane. The aim of the present study was to explore erythrocyte ghost’s proteome to identify changes occurring under the influence of three bee venom peptides-melittin, tertiapin, and apamin. We conducted preliminary experiments on the erythrocyte ghosts incubated with these peptides at their non-hemolytic concentrations. Such preparations were analyzed using liquid chromatography coupled with tandem mass spectrometry. It was found that when higher concentrations of melittin and apamin were used, fewer proteins were identified. Moreover, the results clearly indicated that apamin demonstrates the greatest influence on the RBCs ghosts proteome. Interestingly, the data also suggest that tertiapin exerted a stabilizing effect on the erythrocyte membrane. The experiments carried out show the great potential of proteomic research in the projects focused on the toxin’s properties as membrane active agents. However, to determine the specificity of the effect of selected bee venom peptides on the erythrocyte ghosts, further proteomic research should be focused on the quantitative analysis.

## 1. Introduction

Red blood cells (RBCs), as the most abundant blood morphotic elements, are important cell models for studying the action of membrane-active agents [1]. In a number of works to date, in-depth and extensive proteomic analyses of RBCs have been performed, the purpose of which was to learn the role, organization and composition of the proteome of these cells [2,3,4]. Changes in the structure and function of the red blood cell membrane associated with several diseases and disorders were also investigated [5,6].

Recently, apitherapy has become a promising direction for the introduction of new treatments [7]. Therefore, research focused on discovering new bioactivities of particular bee products, including honey, pollen, propolis, royal jelly and venom, can be important in the field of biotechnology and pharmacology development. In our previous studies, we have demonstrated that the ethanol extracts from Polish propolis are a rich source of phenolic acids and flavonoids and effectively protect RBCs against in vitro oxidative damage [8,9]. Moreover, we have shown that melittin, the major component of venom, modulates the immune activity of insect hemocytes [10].

In the present study, we proposed a proteomic approach to investigate the effects of three main bee venom peptides, melittin, tertiapin, and apamin, on the human RBCs membrane. Although the effects of apitoxin on cells and living organisms have already been studied, their therapeutic value and molecular mechanism of action are still not fully explored, hence the proteomic approach can be a valuable tool in further in-depth analyses.

Melittin is a well-known amphipathic apitoxin composed of 26 amino acids. It exhibits the ability to intercalate into the RBCs membrane [11]. Many studies focused on the interaction between plasmatic membranes and melittin have proven that this peptide is involved in the disruption of phospholipids packaging in the lipid bilayer, channels, and pores formation, membrane protein aggregation, and naturally, lysis [11,12]. Although, in high doses delivered to the human body, melittin is responsible for itching, local reaction, and pain, in a lower concentration it may play a positive, anti-inflammatory role based on the inhibition of phospholipase A2 [12,13,14]. Interestingly, from the pharmacological and biotechnological points of view, the interaction between melittin and cell membranes is responsible for strong antimicrobial properties [15].

The next peptide tested, tertiapin, is not as widely described as melittin or apamin. This neurotoxin consists of 21 amino acids. Tertiapin affects the human body by blocking inward-rectifier potassium channels [15,16]. There are also studies that have confirmed the usefulness of tertiapin for preventing disorders in atrioventricular transmission [16]. However, at present it is mainly described and used as a potassium channel modulator.

Apamin is another bee venom peptide tested. Like tertiapin, apamin belongs to the neurotoxin peptides. It comprises 18 amino acids with two disulfide bonds. Apamin acts mainly in the central nervous system, blocking Ca^2+^-dependent potassium channels [13]. However, apamin activity is not restricted to the grey matter of the brain, but it also locates in the liver and the adrenal cortex [17]. Animal studies have shown that modulation of potassium channels may be a crucial mechanism in the treatment of learning and memory deficits [18,19,20]. Moreover, recent reports suggest a positive effect of the apamin as a treatment for Parkinson’s disease [21,22]. Apamin protects undamaged neurons from degeneration, and also restores the activity of silent neurons [13]. Considering the application of apamin for RBCs storage, it has been pointed out that the addition of this non-hemolytic peptide together with glucose and/or mannitol may extend RBCs storage periods in blood banks without marked structural changes [23].

To the best of our knowledge, there are no studies dealing with the influence of melittin, tertiapin, and apamin on the RBCs membrane proteome. Since matrix-assisted laser desorption/ionization time-of-flight mass spectrometry (MALDI-TOF MS) is proved to be a powerful tool for qualitative analysis, we decided to use it in combination with nano-liquid chromatography (nLC). Therefore, the aim of the current study was to explore human erythrocyte membrane proteome and to point at changes occurring under the influence of bee venom peptides.

## 2. Results

### 2.1. Hemolytic Activity of Apitoxins

**Figure 1:**
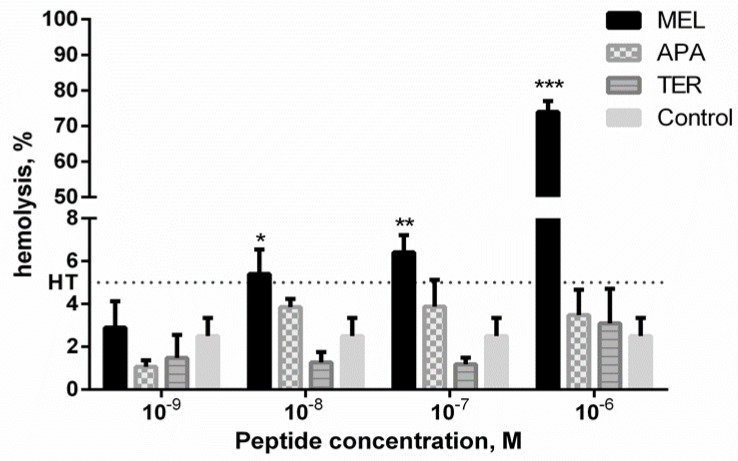
Hemolytic activity (%) of bee venom peptides: melittin (MEL), apamin (APA), and tertiapin (TER) in human erythrocytes after 1 h of incubation at 37 °C. Cropped line is plotted for a hemolysis threshold (HT) of 5%. The hemolysis higher than 5% indicates membrane-perturbing activity of compound studied. Values are presented as the mean ± SEM; *, *p* ≤ 0.05, **, *p* ≤0.01, ***, *p* ≤ 0.01 (statistical analysis was conducted between peptides at the same concentration using two-way ANOVA test). *N* = 12.

Melittin, as a known hemolytic compound, induced concentration-dependent RBCs lysis (Figure 1). Apamin and tertiapin did not show the hemolytic potency in the concentration range used. The degree of hemolysis for the control RBCs was calculated at 2.59 ± 1.40%. Interestingly, the degree of hemolysis in the presence of tertiapin at concentration of 10^−9^–10^−7^ M and apamin at concentration of 10^−9^ M was lower than that of control RBCs (Figure 1). No effect of toxins on discoid RBCs shape was observed at the non-hemolytic concentrations (results are not presented). To study the effect of toxins on RBCs ghosts, the selected sublytic concentrations (hemolysis < 5%) were used.

### 2.2. Red Blood Cells (RBCs) Ghosts Proteome

**Figure 2:**
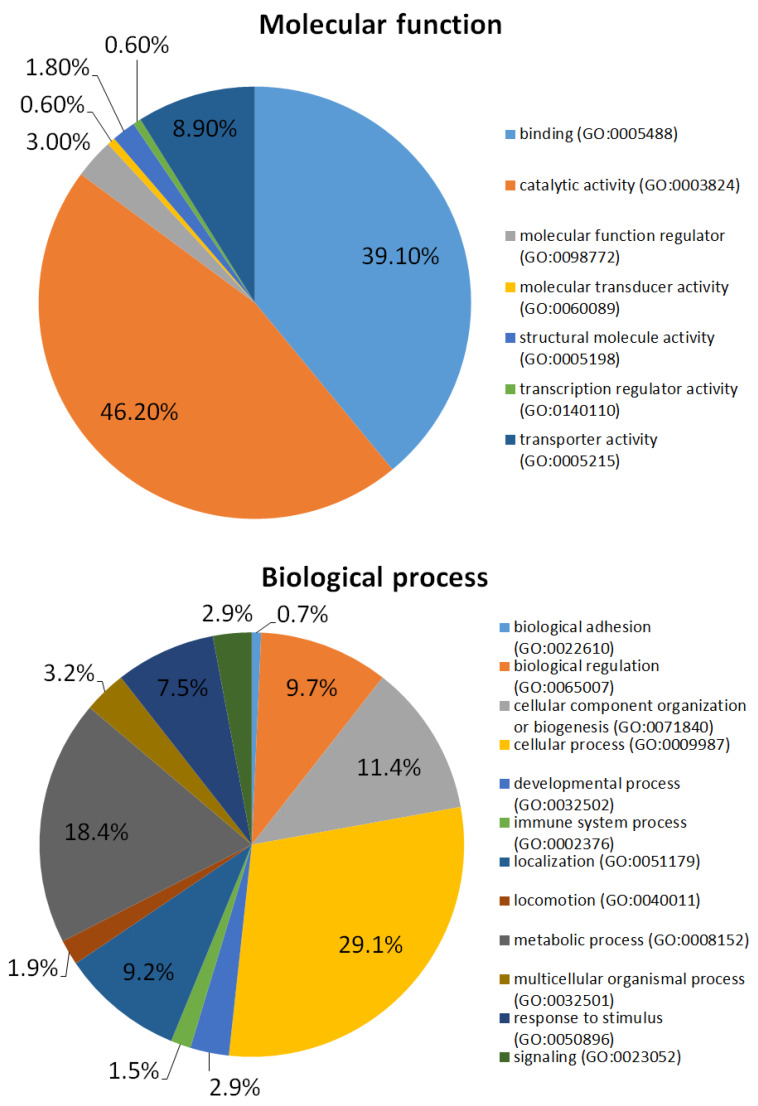
Gene ontology (GO) analysis of the identified RBCs ghosts’ proteins based on PANTHER database including molecular function and biological process (source: https://www.pantherdb.org/).

In total, 248 RBCs ghosts’ proteins were identified based on 1953 peptides (Supplementary Materials, Table S1: List of proteins identified in RBCs). Proteins were categorized using gene ontology analysis (PANTHER database) with regard to molecular function and biological process, as shown in Figure 2. Some of the identified proteins possess a catalytic activity (78 proteins); 66 proteins possess a binding activity. The rest of the proteins are involved in regulatory function, and possess the following activities: molecular transducer, structural molecule, transcription regulator, transporter. Considering biological process RBCs ghosts’ proteins are mostly involved in cellular process (120 proteins), metabolic process (75 proteins) and subcellular localization (38 proteins).

### 2.3. The Influence of Different Concentrations of Bee Venom Peptides on the Human Erythrocyte Membrane Proteome

The first comparison was made to analyze the significance of using different concentrations of bee peptides. It was found that more RBCs ghosts’ proteins were identified in samples with the lower concentration of melittin (10^−9^ vs. 10^−8^ M). The same relationship was found for the samples treated with apamin (10^−9^ vs. 10^−6^ M). Interestingly, there was no significant difference in the number and quality of the identified proteins in the samples treated with different concentrations of tertiapin (10^−9^ vs. 10^−6^ M). Only a few proteins were different, which could be due to the analytical protocol and instrument variations. They were: bleomycin hydrolase (BLMH_HUMAN), Protein S100-A8 (S10A8_HUMAN), Putative deoxyribose-phosphate aldolase (DEOC_HUMAN), S-formylglutathione hydrolase (ESTD_HUMAN), and Selenium-binding protein 1 (SBP1_HUMAN). The results are presented in the Supplementary Materials in Table S2: Differences in protein identification between different melittin concentrations and Table S3: Differences in protein identification between different apamin concentrations.

### 2.4. The Influence of Specific Bee Venom Peptides on the Erythrocyte Membrane Proteome

The second comparison concerned the qualitative analysis of all samples. Therefore, the results obtained for each bee venom peptide (with different concentrations) and controls were combined separately. The data is shown in the Supplementary Materials in Table S4: List of proteins identified in RBCs samples treated with melittin; Table S5: List of proteins identified in RBCs samples treated with apamin; Table S6: List of proteins identified in RBCs samples treated with tertiapin; and Table S7: List of proteins identified in control RBCs samples. The largest changes in the composition of RBCs ghosts proteins were observed in samples treated with apamin. Eleven proteins which were identified in control samples (and also in samples treated with melittin and tertiapin) were not identified in samples treated with apamin, and 8 proteins were only identified in samples with apamin. It should be noted that two of these proteins (ESTD_HUMAN, PA1B3_HUMAN) were only identified with one peptide. In this study, we established the criteria, that precursor ions with a signal-to-noise ratio above 15 were selected for further identification. Therefore, it may be assumed that peptides found below that threshold were either absent in the sample or present in a deficient concentration. Interestingly, apamin induced color changes in the obtained RBCs ghosts, which appeared to be much less pink compared to other samples. Proteins that differed in the apamin-treated sample compared to other samples are presented in the Table 1.

## 3. Discussion

RBCs are undoubtedly a key components of the circulation system and hence the composition and structure of their membrane, including the proteome, have become the subject of a number of analyses [6]. It has been confirmed that the RBCs membrane consists of about 20 major proteins and 850 minor ones [24]. Many different advanced methodologies have been proposed to obtain the best characterization of the RBCs proteome. Several methods for membrane extraction, depletion techniques, fractionation strategies and high-throughput MS analyses can be found in the literature [25]. In this study, a quick and reliable method based on nLC coupled with MALDI-TOF MS was used. It should be emphasized that the proposed methodology enabled the identification of all proteins which form the erythrocyte membrane skeleton, including spectrin, ankyrin, protein 4.1R or actin [26]. Moreover, the vast majority of proteins previously considered as important membrane compounds [27,28] were detected. Therefore, the obtained results are sufficient to do the preliminary assessment of the bee venom peptides activity. The results of GO analysis allowed to the division of identified proteins into specific groups based on their molecular function and biological process. The obtained results are consistent with the observations reported in other studies focused on the RBCs membrane proteome [28].

Despite the potential uses of bee venom and apitoxins in biotechnology and medicine, their action at the cellular level is still not fully understood [29]. In this study, the effects of three main bee venom compounds: melittin, tertiapin and apamin, on the RBCs ghost’s proteome were evaluated. It should be noted that the hemolytic properties of the bee venom compounds, especially melittin, as well as venom-induced cell lysis, have already been extensively explored [30]. Therefore, non-hemolytic concentrations of the analyzed peptides were used in this study. The differences occurring between the action of these venom peptides were evaluated based on the qualitative analysis. Changes in the composition of the proteins, may indicate transfer of proteins into the incubation solution or non-specific degradation of proteins under the influence of the used apitoxins. 

Melittin, due to its interaction with phospholipids, can act as a natural surfactant, leading to disruption of the lipid bilayer [30]. In this study, no significant remodeling of the RBCs membrane proteome was observed after melittin treatment at concentrations of 10^−9^ M and 10^−8^ M compared to the control samples. However, there were some differences in the results obtained by using these two concentrations, which indicate that when a higher concentration was used, a smaller number of proteins was identified. Therefore, these results may suggest that even small doses of melittin can lead to alteration in the RBCs ghost’s proteome. Treatment with a higher concentration of melittin resulted in a lack of identification of proteins in RBCs involved in basic cellular processes (Supplementary Materials, Table S2: Differences in protein identification between different melittin concentrations). These proteins, including aldolase, metalloprotease, GTPase, and acyltransferase were mainly involved in enzymatic processes. Moreover, some of them are parts of the signal transduction pathway. Thus, melittin can cause a significant disruption of RBCs functioning. These results showed that in-depth analysis of proteomes can be an important source of knowledge about the effects of melittin on the human body.

So far, no interaction between the cell membrane and tertiapin has been observed. Our results confirmed that this bee venom peptide does not influence the RBCs ghosts proteome at the concentrations used. Despite the use of different concentrations (10^−9^ M and 10^−6^ M), the results of protein identification were similar for both the doses and for the control samples. In addition, in the presence of tertiapin in the concentration range 10^−9^–10^−7^ M, RBCs had a lower degree of hemolysis compared to the control (Figure 1). Therefore, it might be suggested that tertiapin had a stabilizing effect on the structure of the RBCs membrane. In the literature, there are only a few uses of tertiapin for therapeutic purposes described, mainly based on its ability to block potassium channels [16,31,32]. Our results are consistent with previous studies concerning physiological action of tertiapin, which revealed that this compound was non-toxic in intravenous injection [33]. However, Miroshnikov et al. [34] showed that tertiapin may interact with calmodulin, which leads to inhibition of the enzyme-activating action of calmodulin. Current studies on the role of calmodulin suggest that, due to its interaction with several RBCs proteins, such as the spectrin and Ca^2+^/ATPase calcium pump, it is required to maintain normal erythrocytes morphology [35]. For these reasons, further studies are needed, especially regarding quantitative analysis of the RBCs membrane proteome.

Our results suggest that apamin affects the RBCs membrane. There may be several reasons for differences in protein identification between controls and with samples treated with melittin and tertiapin, and samples treated with apamin. Firstly, apamin may stabilize and protect some of the RBCs proteins during applied sample preparation. In the proteome of RBCs treated with apamin at concentration of 10^−6^ M, we did not identify the calpain, protein which was found in other samples. It was shown that inhibition of this protein protected erythrocyte membrane-associated cytoskeletal proteins from proteolytic degradation which occurred when the cells were rendered permeable to Ca^2+^ [36]. This may suggest that proteins do not penetrate into the incubation solution or/and they are protected from nonspecific degradation. As a consequence, some proteins that were not detected in the control samples appeared in the samples treated with apamin. It should be taken into account that even small changes in the protein quantities may affect the identification process and the measuring capabilities of the instrument. In MALDI-MS, one of the difficulties is that the analyte signals can be suppressed by signals derived from other molecules or compounds in the sample [37]. Moreover, the reproducibility of MALDI-TOF results depends significantly on the homogeneity of the co-crystals between matrix and analyte. Changes in external factors like temperature, pressure, and humidity may influence the co-crystallization step and cause day-to-day variation [38]. Therefore, the next step in this research should be a quantitative analysis of the proteome.

Due to its potential application in biotechnology and medicine, apamin seems to be one of the most important bee venom peptides. To date, studies on the activity of this peptide have been largely focused on its effect on the central nervous system [13,19,23,39,40]. Interestingly, apamin as a K^+^ channel blocker agent, has also been applied as a compound to preserve of whole blood as well as a suspension of red blood cells in the blood bank [23]. It was indicated that apamin extends periods of storage time of blood preventing in vitro RBCs aging. Our research also confirmed the significant influence of apamin on the RBCs. Forty-seven protein compounds were detected only in samples treated with apamin at concentration of 10^−9^ M compared to concentration of 10^−6^ M (Supplementary Materials, Table S3: Differences in protein identification between different apamin concentrations). These identified proteins exhibit mainly enzymatic activity with regulatory functions. Most of them participate in cellular metabolism and degradation of unneeded compounds. Therefore, especially at the lower concentration used, apamin is probably responsible for the membrane stabilization, as was demonstrated in the hemolytic assay (Figure 1, degree of hemolysis at 10^−9^ vs. 10^−6^ M). In the proteome of apamine-treated RBCs, membrane inhibitor of reactive lysis protein (CD59) also known as protectin was detected (Table 1). This protein functions as an inhibitor of the complement membrane attack complex by preventing the incorporation of the multiple copies of the complement component 9 (C9) required for complete formation of the osmolytic pores in the cell membrane [41]. The absence of the T-complex protein 1 in samples in which hemolysis occurred may also support this hypothesis. This protein is a component of the chaperonin-containing T-complex (TRiC), a molecular chaperone complex that helps in folding proteins after ATP hydrolysis. The TRiC complex may also play a role in actin and tubulin [42]. Additionally, differences in abundance of a decay-accelerating factor (CD55) were detected in samples treated with apamin. It functions to protect RBCs from complement-mediated lysis by inhibiting the aggregation of some convertases of the classical and alternative pathways, and thereby regulates the complement cascade [43,44]. Apamin may also affect the RBC’s membrane composition. We detected two proteins-CD44 and Basigin (BSG)—the first being major hyaluronan receptor [45] and the second associated with monocarboxylate transporters (MCTs) [46]. It was shown that BSG served as a chaperone required for the plasma membrane translocation of MCTs [46] which catalyzed the transport of substituted short-chain fatty acids, lactate, pyruvate and ketone bodies, across the plasma membrane. BSG triggers also the formation of a lipid raft-associated supramolecular complex which helps to function MCTs and hyaluronan appears to stabilize and enhance its activity [47]. However, further studies, including proteomics, are needed to extend the possibilities to use apamin in clinical applications.

To summarize, the performed analysis allowed for examination of the apitoxin peptides melittin, tertiapin and apamin as membrane active-agents and their particular impact on the RBCs membrane proteome. The conducted studies show the huge potential of proteomic analysis in the projects focused on the toxins activity and the results obtained clearly indicated that apamin exerts the greatest influence on the RBCs proteome. Moreover, changes in the RBCs membrane proteome may be associated with the stabilizing action of apamin on this structure. Interestingly, despite the potential influence of tertiapin on calmodulin, and thus on the structure of the erythrocyte membrane, we did not observe any qualitative changes induced in the membrane by this peptide. For these reasons, the next step will be a quantitative analysis of the RBCs ghost’s proteome supplemented by evaluation of the synergistic action of tested compounds of apitoxin. Probably the planned analysis will allow to fully understand all the changes observed in RBCs membrane, especially after apamin and tertiapin treatment. This knowledge may be very useful, especially through the prism of usage of these peptides as cell membrane stabilizers.

## 4. Materials and Methods 

### 4.1. Reagents

Phosphate-buffered saline (PBS) buffer compounds (NaCl, KCl, Na_2_HPO_4_, and KH_2_PO_4_), glucose, glutaraldehyde, paraformaldehyde, glycerol, *N*-ethylmaleimide, were purchased from Avantor Performance Materials Poland S.A. (Gliwice, Poland). Ammonium phosphate monobasic, dithiothreitol (DTT) iodoacetamide, and trifluoroacetic acid (TFA) were supplied by Sigma Aldrich (St. Louis, MO, USA), and α-cyano-4-hydroxycinnamic acid (HCCA) was supplied by Bruker Daltonics (Bremen, Germany). Trypsin was supplied by Promega (Madison, WI, USA) Acetonitrile (ACN), 2-propanol, ethanol, and acetone were supplied by J.T. Baker (Center Valley, PA, USA). The reagents were of analytical grade or better. The water used in the study was of Milli-Q quality. Melittin, tertiapin and apamin were supplied by Sigma Aldrich (St. Louis, MO, USA).

### 4.2. Samples Preparation

#### 4.2.1. Human Erythrocyte

Fresh human red blood cells (RBCs) concentrates (hematocrit ~ 65%) were purchased from the blood bank in Poznań according to the bilateral agreement no. ZP/907/1002/18. RBCs were washed three times (960× *g*, 10 min, 4 °C) (Sigma 3–30K Sartorius AG, Göttingen, Germany) in 7.4 pH phosphate buffered saline (PBS—137 mM NaCl, 2.7 mM KCl, 10 mM Na_2_HPO_4_, 1.76 mM KH_2_PO_4_) supplemented with 10 mM glucose. After washing, RBCs were suspended in the PBS buffer at 1.65 × 10^9^ cells/mL, stored at 4 °C and used within 5 h.

#### 4.2.2. Hemolysis Assay

Cytotoxic effect of toxins was determined by the hemolytic assay as described before [48]. RBCs (1.65 × 10^8^ cells/ml, 1.5% hematocrit) were incubated in PBS (7.4 pH) supplemented with 10 mM glucose and containing melittin (10^−9^–10^−5^ M), tertiapin or apamin (10^−9^–10^−6^ M) for 1 hour at 37 °C in a thermo-shaker (BioSan Thermo-Shaker TS-100C, Biosan, Riga, Latvia). Samples with RBCs incubated in PBS without toxins were taken as the controls. Each sample was repeated three times, and the experiments were repeated four times with RBCs from different blood donors. Following incubation, the RBCs suspensions were centrifuged (Sigma 3–30 K Sartorius AG, Göttingen, Germany) (960× *g*, 10 min, 4 °C) and the released hemoglobin was determined spectrophotometrically at 540 nm (absorption A) in an EPOLL2000 ECO spectrophotometer (PZ EMCO, Warsaw, Poland). The absorption corresponding to a complete hemolysis (absorption B) was acquired after the centrifugation of RBCs treated with ice-cold distilled water. The percentage of hemolysis was then calculated (% hemolysis = value of absorption A/value of absorption B × 100). The results were expressed as percentage of hemolysis (%). The non-hemolytic concentrations of every peptide were selected for the next step of the study. The statistical analysis of obtained hemolysis data was conducted using GraphPad software version 6 (GraphPad Software, San Diego, CA, USA) (licensed by the Department of Animal Physiology and Development, AMU). The analysis was conducted between peptides at the same concentration using a two-way ANOVA test with Tukey’s post hoc. Values of *p* ≤ 0.05 (*), *p* ≤ 0.01 (**) or *p* ≤ 0.001 (***) were considered statistically significant.

#### 4.2.3. Erythrocytes Shape Evaluation

RBCs (1.65 × 10^8^ cells/mL, 1.5% hematocrit) were incubated in PBS (7.4 pH) supplemented with 10 mM glucose and containing melittin, tertiapin or apamin (10^−9^–10^−6^ M) for 1 hour at 37 °C in a thermo-shaker (BioSan Thermo-Shaker TS-100C, Biosan, Riga, Latvia). Samples with RBCs incubated in PBS without toxins were taken as the controls. Each sample was repeated three times, and the experiments were repeated four times with erythrocytes from different blood donors. Following incubation, RBCs were fixed with 5% paraformaldehyde plus 0.01% glutaraldehyde for 1 h at room temperature (RT). Following washing, the RBCs were settled on polylysine-treated (0.1 mg/mL, 10 min) cover glasses and then washed. The cells were mounted on 80% glycerol. The coverslips were sealed with nail polish. A large number of RBCs in several separate experimental samples were studied using RED-233 MOTIC (Conbest, Cracow, Poland) light microscope (100 × immersion oil objective, 10 × ocular). The RBCs shape was described according to the Bessis classification [49,50].

#### 4.2.4. Erythrocytes Ghosts

RBCs ghosts were prepared in hypotonic PBS at pH 7.4 according to the method by Dodge [49]. Briefly, RBCs were incubated (1.65 × 10^8^ cells/ml, 1.5% hematocrit) in EP vials for 15 min at RT with 100 mM N-ethylmaleimide (NEM) in hypotonic (20 mM) PBS phosphate buffer. Following incubation, erythrocytes were re-suspended in 5 mM PBS on ice for the next 20 min. After incubation, RBCs ghosts were centrifuged (17,000× *g*, 20 min, 4 °C) and washed 3 times (17,000× *g*, 20 min, 4 °C) with 5 mM phosphate buffer supplemented with 100 mM NEM. Finally, RBCs ghosts were washed in isotonic PBS (17,000× *g*, 20 min, 4 °C). The toxins were added to the ghost suspension in isotonic PBS buffer (pH 7.4) to get the selected non-hemolytic concentrations: for melittin 10^−9^ and 10^−8^ M, for apamin and tertiapin 10^−9^ and 10^−6^ M, respectively. Samples with RBCs ghosts incubated in PBS without toxins were taken as the controls. EP vials were incubated for 1 h at 37 °C in a thermo-shaker (BioSan Thermo-Shaker TS-100C, Biosan, Riga, Latvia). Each sample was repeated three times. Following incubation, samples were washed three times in isotonic PBS (17,000× *g*, 20 min, 4 °C), re-suspended in the distilled cold water and stored at −80 °C until proteome analysis.

### 4.3. nLC-MALDI-TOF/TOF MS/MS Analysis

RBCs ghosts were digested with trypsin (Promega, Madison, WI, USA) using modified protocol from pierce in-solution tryptic digestion kit. In brief, proteins were denatured, reduced, and alkylated. Then trypsin was added and samples were digested in 37 °C overnight. Obtained tryptic peptides were further separated using nLC and obtained fractions were analyzed using MALDI-TOF/TOF MS/MS. nLC platform contained EASY-nanoLC II system (Bruker Daltonics, Germany), fraction collector Proteineer-fc II (Bruker Daltonics, Germany) and HyStar software version 3.2. Detailed information about trap column and separation column are presented in our previous paper [51]. Both mobile phases–A (water) and B (90% acetonitrile), contained 0.05% of trifluoroacetic acid. The gradient of elution was 2–50% of mobile phase B during 96 min with the flow rate 300 nL/min. The separation process allowed to obtain 384 fractions of each sample, which were automatically mixed with the α-cyano-4-hydroxycinnamic acid matrix solution. Eluents were spotted onto the MALDI plate (MTP AnchorChip 384, Bruker Daltonics, Germany). Subsequently, collected fractions were analyzed using tandem mass spectrometry-MALDI-TOF/TOF (UltrafleXtreme, Bruker Daltonics, Germany). Typical settings of the mass spectrometer are described in detail in our previous paper [52]. Analyses were performed in the mass range of 700–3500 Da. Peptide Calibration mixture (Bruker Daltonics, Germany) was used for external calibration of each MS spectrum. Precursor ions with signal-to-noise ratio above 15 were selected for further identification. Several software were used for tandem mass spectrometry analysis, data acquisition, and evaluation: WARP LC 1.3, ProteinScape 3.1, FlexControl 3.4 and FlexAnalysis 3.4. (Bruker Daltonics, Germany). Protein identification was conducted using BioTools 3.2 (Bruker Daltonics, Germany), the SwissProt database and Mascot 2.4.1 search engine (Matrix Science, London, UK). The following search parameters were used: taxonomy restriction: “Homo sapiens”; enzyme: trypsin; peptide mass tolerance: 25 ppm; MS/MS fragment mass tolerance: 0.7 Da; peptide charge: 1+; monoisotopic mass. Each sample was analyzed in triplicate. Results were analyzed using PANTHER (protein annotation through evolutionary relationship) Classification System database (http://pantherdb.org.) [53]. Moreover, results derived from erythrocytes membrane treated with different bee venom peptides (and different peptides concentrations) were compared. During the comparisons different proteins isoforms or homologs were considered as the same protein (for example: Proteasome subunit beta type-6 and Proteasome subunit beta type-7). To obtain the most reliable results, only proteins identified based on two peptides with score above 80 and false discovery rate under 1%, should be considered. However, in our comparisons we presented and described proteome changes only, when none single peptide from the specific protein was present. Even when there was only one peptide with score under 80 observed, there is no certainty whether it is absent or present.

## 5. Conclusions

The presented research results have shown that the nLC-based method in combination with MALDI-TOF MS can guarantee a quick and accurate analysis of the RBCs ghost proteome. Therefore, this method will also be developed and adapted to the analysis of insect hemocytes. This is related to the fact that our previous studies have shown a similar lytic effect of melittin on the hemocytes of the *Tenebrio molitor* beetle [10], like in case of human erythrocytes. The continuation of research on insect cells is important due to searching for a new model organism in the toxicology studies.

## Figures and Tables

**Table 1:** Proteins identified and unidentified in red blood cells’ (RBCs) ghosts treated with apamin (10^−9^ and 10^−6^ M).

Proteins not Detected in the RBCs Ghosts Samples Treated with Apamin			Proteins Identified only in Rbcs Ghosts Samples Treated with Apamin		
No.	Accession	Protein	No.	Accession	Protein
1.	SBP1_HUMAN	Selenium-binding protein 1	1.	BASI_HUMAN	Basigin
2.	TGM2_HUMAN	Protein-glutamine gamma-glutamyltransferase 2	2.	S29A1_HUMAN	Equilibrative nucleoside transporter 1
3.	ESTD_HUMAN	S-formylglutathione hydrolase	3.	CD44_HUMAN	CD44 antigen
4.	F10A1_HUMAN	Hsc70-interacting protein	4.	KAP0_HUMAN	cAMP-dependent protein kinase type I-alpha regulatory subunit
5.	LDHB_HUMAN	L-lactate dehydrogenase B chain	5.	ABCB6_HUMAN	ATP-binding cassette sub-family B member 6, mitochondrial
6.	BLMH_HUMAN	Bleomycin hydrolase	6.	CD99_HUMAN	CD99 antigen
7.	NSF1C_HUMAN	NSFL1 cofactor p47	7.	BCAM_HUMAN	Basal cell adhesion molecule
8.	S10A8_HUMAN	Protein S100-A8	8.	CD59_HUMAN	CD59 glycoprotein
9.	RADI_HUMAN	Radixin			
10.	NDKA_HUMAN	Nucleoside diphosphate kinase A			
11.	PA1B3_HUMAN	Platelet-activating factor acetylhydrolase IB subunit gamma			

## Supplementary Materials

The following are available online at https://www.mdpi.com/2218-1989/10/5/191/s1, Table S1: List of all proteins identified in RBCs, Table S2: Differences in protein identification between different melittin concentrations, Table S3: Differences in protein identification between different apamin concentrations, Table S4: List of proteins identified in RBCs samples treated with melittin, Table S5: List of proteins identified in RBCs samples treated with apamin, Table S6: List of proteins identified in RBCs samples treated with tertiapin, and Table S7: List of proteins identified in control RBCs samples.
