# Supplementary material for: The Effect of Bee Venom Peptides Melittin, Tertiapin, and Apamin on the Human Erythrocytes Ghosts: A Preliminary Study
Source: Metabolites. 2020 May 13;10(5):191. doi: 10.3390/metabo10050191 (PMC7281017; doi:10.3390/metabo10050191)
Supplement: Supplementary file 1 [file metabolites-10-00191-s001.zip › Supplementary files - revised/Table S2. Differences in protein identification between different melittin concentrations.docx]

**Table S2.** Differences in protein identification between different melittin concentrations**.**

| **Proteins identified in sample treated with melittin concentration 10^-8^ [M]** | |
| --- | --- |
| **Accession** | **Protein** |
| F10A1_HUMAN | Hsc70-interacting protein |
| NDKB_HUMAN | Nucleoside diphosphate kinase B |
| ALBU_HUMAN | Serum albumin |
|  | |
| **Proteins identified in sample treated with melittin concentration 10^-9^ [M]** | |
| **Accession** | **Protein** |
| CAND1_HUMAN | Cullin-associated NEDD8-dissociated protein 1 |
| FLOT1_HUMAN | Flotillin-1 |
| FLOT2_HUMAN | Flotillin-2 |
| ALDOA_HUMAN | Fructose-bisphosphate aldolase A |
| KELL_HUMAN | Kell blood group glycoprotein |
| AGO2_HUMAN | Protein argonaute-2 |
| TGM2_HUMAN | Protein-glutamine gamma-glutamyltransferase 2 |
| DEOC_HUMAN | Putative deoxyribose-phosphate aldolase |
| RAP1B_HUMAN | Ras-related protein Rap-1b |
| SBP1_HUMAN | Selenium-binding protein 1 |
| YA047_HUMAN | Uncharacterized protein LOC388588 |
