# Supplementary material for: The Effect of Bee Venom Peptides Melittin, Tertiapin, and Apamin on the Human Erythrocytes Ghosts: A Preliminary Study
Source: Metabolites. 2020 May 13;10(5):191. doi: 10.3390/metabo10050191 (PMC7281017; doi:10.3390/metabo10050191)
Supplement: Supplementary file 1 [file metabolites-10-00191-s001.zip › Supplementary files - revised/Table S3. Differences in protein identification between different apamin concentrations.docx]

**Table S3.** Differences in protein identification between different apamin concentrations.

| **Proteins identified in sample treated with apamin concentration 10^-6^ [M]** | | |
| --- | --- | --- |
| **Accession** | | **Protein** |
| KAP0_HUMAN | | cAMP-dependent protein kinase type I-alpha regulatory subunit |
|  | | |
| **Proteins identified in sample treated with apamin concentration 10^-9^ [M]** | | |
| **Acccession** | | **Protein** |
| 1433B_HUMAN | | 14-3-3 protein beta/alpha |
| ACLY_HUMAN | | ATP-citrate synthase |
| ACPH_HUMAN | | Acylamino-acid-releasing enzyme |
| PUR8_HUMAN | | Adenylosuccinate lyase |
| A16A1_HUMAN | | Aldehyde dehydrogenase family 16 member A1 |
| BCAM_HUMAN | | Basal cell adhesion molecule |
| PMGE_HUMAN | | Bisphosphoglycerate mutase |
| CD99_HUMAN | | CD99 antigen |
| CAN1_HUMAN | | Calpain-1 catalytic subunit |
| CAH1_HUMAN | | Carbonic anhydrase 1 |
| CLH1_HUMAN | | Clathrin heavy chain 1 |
| DAF_HUMAN | | Complement decay-accelerating factor |
| CAND1_HUMAN | | Cullin-associated NEDD8-dissociated protein 1 |
| HEM2_HUMAN | | Delta-aminolevulinic acid dehydratase |
| BLVRB_HUMAN | | Flavin reductase (NADPH) |
| GMPR1_HUMAN | | GMP reductase 1 |
| GBB1_HUMAN | | Guanine nucleotide-binding protein G(I)/G(S)/G(T) subunit beta-1 |
| PUR6_HUMAN | | Multifunctional protein ADE2 |
| PSME1_HUMAN | | Proteasome activator complex subunit 1 |
| PSME2_HUMAN | | Proteasome activator complex subunit 2 |
| PSMF1_HUMAN | | Proteasome inhibitor PI31 subunit |
| PSA1_HUMAN | | Proteasome subunit alpha type-1 |
| PSA2_HUMAN | | Proteasome subunit alpha type-2 |
| PSA3_HUMAN | | Proteasome subunit alpha type-3 |
| PSA5_HUMAN | | Proteasome subunit alpha type-5 |
| PSA6_HUMAN | | Proteasome subunit alpha type-6 |
| PSA7_HUMAN | | Proteasome subunit alpha type-7 |
| PSB1_HUMAN | | Proteasome subunit beta type-1 |
| PSB2_HUMAN | | Proteasome subunit beta type-2 |
| PSB3_HUMAN | | Proteasome subunit beta type-3 |
| PSB4_HUMAN | | Proteasome subunit beta type-4 |
| PSB5_HUMAN | | Proteasome subunit beta type-5 |
| PSB6_HUMAN | | Proteasome subunit beta type-6 |
| DDI2_HUMAN | | Protein DDI1 homolog 2 |
| PNPH_HUMAN | | Purine nucleoside phosphorylase |
| DEOC_HUMAN | | Putative deoxyribose-phosphate aldolase |
| AL1A1_HUMAN | | Retinal dehydrogenase 1 |
| PRPS1_HUMAN | | Ribose-phosphate pyrophosphokinase 1 |
| TCPA_HUMAN | | T-complex protein 1 subunit alpha |
| TCPB_HUMAN | T-complex protein 1 subunit beta | |
| TCPD_HUMAN | T-complex protein 1 subunit delta | |
| TCPE_HUMAN | T-complex protein 1 subunit epsilon | |
| TCPH_HUMAN | T-complex protein 1 subunit eta | |
| TCPG_HUMAN | T-complex protein 1 subunit gamma | |
| TCPQ_HUMAN | T-complex protein 1 subunit theta | |
| TERA_HUMAN | Transitional endoplasmic reticulum ATPase | |
| RD23A_HUMAN | UV excision repair protein RAD23 homolog A | |
